# Oral and Pharyngeal Diverticula: A Rare Case of Dysphagia and Diagnostic Challenges

**DOI:** 10.3390/medicina60101628

**Published:** 2024-10-05

**Authors:** Abdullah Mohammed Alfaris, Nisreen Naser Al Awaji, Shaden Abdulmohsen Alabdulkarim, Ammar Mammoun Mallisho, Anas Osman Hamdoun, Samir Mohammed Bawazir, Noura Ahmed AlAjroush

**Affiliations:** 1 Rehabilitation Department, King Abdullah Bin Abdulaziz University Hospital, P.O. Box 7 47330, Riyadh 11552, Saudi Arabia; amalfaris@kaauh.edu.sa (A.M.A.); saalabdulkarim@kaauh.edu.sa (S.A.A.); 2 Department of Health Communication Sciences, College of Health and Rehabilitation Sciences, Princess Nourah Bint Abdulrahman University, P.O Box 84428, Riyadh 11671, Saudi Arabia; nnalawaji@pnu.edu.sa; 3 Medical Imaging Department, King Abdullah Bin Abdulaziz University Hospital, P.O. Box 7 47330, Riyadh 11552, Saudi Arabia; ammallisho@kaauh.edu.sa; 4 Radiology Department, King Abdullah Bin Abdulaziz University Hospital, P.O. Box 7 47330, Riyadh 11552, Saudi Arabia; aohamdoun@kaauh.edu.sa; 5 Pediatric ORL Department, Prince Sultan Military Medical City, Riyadh 12233, Saudi Arabia; smbawazir@psmmc.med.sa

**Keywords:** oral diverticulum, pharyngeal diverticulum, dysphagia, buccal pouches

## Abstract

This report describes the case of a 62-year-old male patient in Saudi Arabia who developed a diverticular pouch in his oral cavity. Diverticula are rare conditions that appear as protrusions or pouches within the digestive tract’s inner lining. The condition can occur in different parts of the digestive system, but the colon is the most affected part. As part of the patient’s symptoms, he presented with dysphagia, weight loss, and globus sensations. Multiple diagnostic tests, including ultrasound, computerized tomography (CT), and magnetic resonance imaging (MRI), did not identify the diverticula. Barium and upper gastrointestinal studies were the diagnostic tests that provided accurate diagnoses where several diverticula were discovered during the videofluoroscopic swallow study (VFSS) and fiberoptic endoscopic evaluation of swallowing (FEES). Surgical intervention was recommended by the multidisciplinary team; however, the patient rejected this treatment option. This report highlights the necessity for instrumental swallowing diagnostic evaluation in the workup of the infrequent etiologies of dysphagia and suggests a gap in the current knowledge, prompting further studies on the etiology, incidence, and optimum management of upper gastrointestinal (GI) tract diverticular disease.

## 1. Introduction

A diverticulum is a protrusion or pouch in the digestive tract’s inner lining that forms into a small pouch with a narrow neck due to the deficiency of outer muscle covering [1,2]. Diverticula can be categorized as unilateral or bilateral, acquired or congenital, and true or false. False diverticula do not involve the muscular layers or adventitia, whereas true diverticula involve each layer of the structure, including the muscular propria and adventitia [2]. This condition can affect various parts of the GI tract, including oral and pharyngeal structures. Even though multiple studies have revealed that diverticula occur more frequently in the colon, they are rarely located in the oral cavity [3,4]. Numerous studies have shown that factors such as a low-fiber diet, obesity, inactivity, and various medications may contribute to the presence of a pouch or diverticula [1,5,6]. Age is a key factor in the prevalence of diverticula, with a significant increase in individuals above the age of 60 years, affecting men and women equally [2,4,6,7].

Bedside Swallowing Assessment (BSA), a non-instrumental procedure, is usually the first step for assessing patients with swallowing difficulties. BSA includes obtaining case history, Oral Motor Examination (OME), and swallowing assessment using various bolus sizes and consistencies whilst observing for signs of oral and pharyngeal dysphagia from bolus control to signs of aspiration [8]. Instrumental assessment, such as VFSS or FEES, is the golden standard for assessing all patients [9,10]. VFSS is usually conducted in the X-ray department in a hospital where different consistencies of food and different bolus sizes are mixed with barium (or other contrast substances). This enables the clinician to view the bolus under X-ray, assessing the anatomy and physiology of the oropharyngeal cavity in addition to the presence, timing, and amount of penetration and aspiration [9]. In FEES, the patient’s swallowing is assessed via inserting a fiberoptic tube with a camera through the nose whilst the patient is swallowing different bolus sizes and consistencies of food [9]. However, performing either procedure is not always possible as the expertise and equipment required are not always readily available [11]. Moreover, other factors such as radiation exposure to VFSS, the patient’s overall general health (i.e., medically unstable), the invasiveness nature of both procedures, the patient’s consent, cost and time restrictions, and the patient’s contact precaution and cognitive dysfunction contraindicate the use of instrumental swallowing assessment [11,12]. Nonetheless, they are a necessity for cases when silent aspiration or other anatomical etiologies are suspected. Due to the rarity of upper GI tract diverticula, clinicians are less likely to consider it as the cause of the patient’s symptoms [2,4,13,14].

The majority of diverticular cases are asymptomatic [7,14]; however, some patients with diverticula may present with a range of symptoms, including dysphagia as the primary symptom, and other complications such as neck pain, weight loss, regurgitation, dysphonia, a globus sensation, or a neck mass in addition to a range of other upper gastrointestinal tract issues [7,14]. These signs and symptoms depend on the size of the diverticulum, whether the pouch drains easily, and whether it is infected [15,16,17,18]. The diagnosis of this condition can be effectively established via barium studies [17,18,19]. For patients with diverticulum/diverticula, a study in 2018 has shown that the most effective treatment is surgical via an endoscopic or an external cervical approach [5].

Currently, there are only a few case reports and studies on this rare occurrence of a diverticular pouch in the oral cavity, none of which are in Saudi Arabia [2,4]. In general, there is insufficient literature about the incidence, prevalence, and severity of this medical condition. This paper discusses a rare case of oropharyngeal diverticula in a male patient who experienced multiple dysphagia symptoms, malnutrition, and weight loss.

## 2. Detailed Case Description

1. Medical History:

A 62-year-old male patient, who is a heavy smoker with a known medical history of diabetes mellitus type 2, hypertension, dyslipidemia, and major depression disorder. He also had a history of laparoscopic cholecystectomy five years ago and of laparoscopic sleeve gastrectomy four years ago. The patient was on Nexium, escitalopram 20 mg, quetiapine 100–200 mg, and aspirin. He presented to the general surgical clinic complaining of difficulties in swallowing food of all consistencies for the past year, which was worsening with time. He described his condition as tasting blood when swallowing, and a bloodstain was seen on his pillow after waking up. He also reported feeling a globus sensation and a looping sensation in his entire oral and pharyngeal cavity and sometimes even in his face. The patient also reported having frequent symptoms of heartburn and bad breath. He denied having any fever, chest infection, joint pain, or diarrhea.

2. First Clinical examination:

Upon clinical examination, the patient underwent the following:

- Thyroid ultrasound (US Thyroid) presented unremarkable results with a normal thyroid size and no palpable nodules.
- CT scan of the brain and neck revealed unremarkable results: no acute brain abnormality, no mass lesion, and no pathologically enlarged lymph nodes.
- Brain MRI showed unremarkable results with no acute brain insult and no brain parenchymal mass lesion. The patient also reported that he had undergone many other clinical examinations and radiographic studies in other hospitals prior to his referral to our clinic, all of which revealed unremarkable results.
- An upper GI study by the radiologist revealed two pockets filled with contrast at the base of the tongue anterior to the pharynx. Another two filling contrast pockets were seen posteriorly at the level of the valleculae, suggestive of pharyngeal diverticula. The mild dilatation of the valleculae was seen with some irregularities and residual contrast, as well as contrast material penetration and mild gastroesophageal reflux.

3. Referral and Further Examination:

The patient was subsequently referred to an Ear, Nose, and Throat (ENT) consultant, who performed a fiberoptic endoscopy, revealing a pouch in his buccal area on both sides. Given the multifaceted presentation of the symptoms, the patient was referred to speech and language pathologists (SLPs) for swallowing assessment.

**Figure 1:**
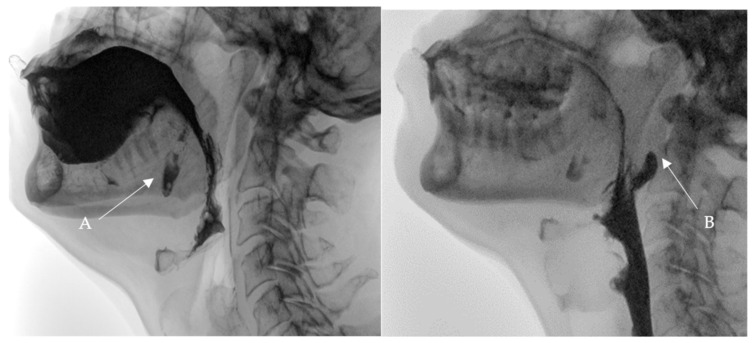
Selected still images from VFSS, lateral view: bolus pooling and residual contrast in (**A**). buccal diverticula and (**B**). pharyngeal diverticulum.

**Figure 2:**
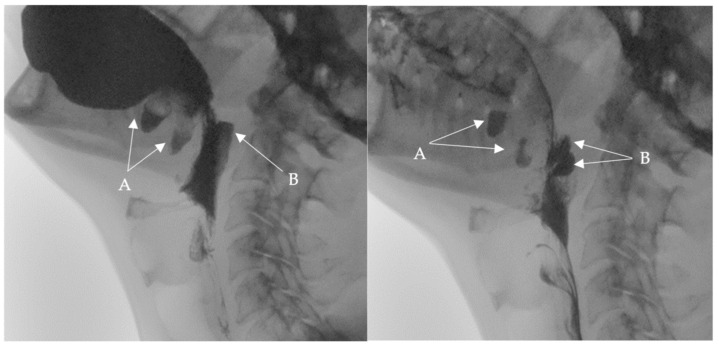
Selected still images from VFSS, lateral view: simultaneous bolus pooling and contrast residue in (**A**) two buccal and (**B**) two pharyngeal diverticula.

4. Swallowing Assessment and Instrumental Evaluation:

At the swallowing clinic, the following assessments were conducted:

- BSA including an OME was conducted and revealed functional swallowing skills. There was no evidence of motor or sensory facial deficits or oral cavity deficits. However, a negative gag reflex was observed.
- A videofluoroscopic swallowing study (VFSS) was conducted to assess the oral, pharyngeal, and esophageal stages of swallowing with lateral and anteroposterior views using liquids of thin, thick, and pureed consistencies. The study, conducted by an SLP and a radiologist, showed two posterior inferior oral pouches (buccal diverticula) clearly seen on the floor of the mouth after swallowing. Moreover, two posterior pharyngeal wall pouches (diverticula) in the oropharynx at the level of the second cervical vertebra were visualized (Figure 1 and Figure 2) in addition to regurgitation and flaccid epiglottis. However, no aspiration or penetration was seen during the study.

5. Subsequent Fiberoptic Endoscopic Evaluation of Swallowing (FEES):

The FEES procedure was conducted with different consistencies of liquid and purée of 1 mL, 3 mL, 5 mL, and 10 mL applied with a teaspoon and a tablespoon. The results indicated the following:

- Oropharyngeal dysphagia characterized by the following features: flaccid uvula and delayed swallowing trigger at the level of the vallecular with thin liquid, thick liquid, and puree.
- Deep residue in vallecular and pyriform sinuses with all the consistencies (suggestive of a vallecula pouch), and signs of reflux.
- No aspiration or penetration with all the consistencies was observed during the study.

**Figure 3:**
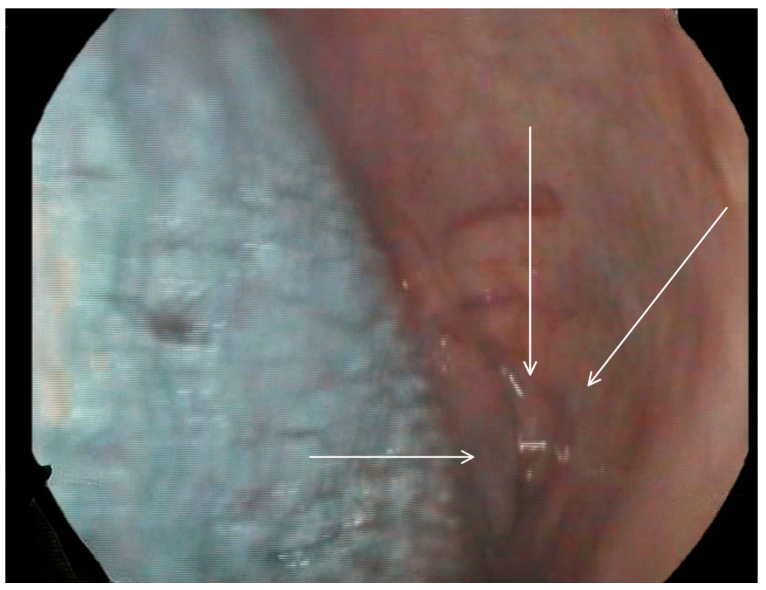
Selected still image captured during the FEES assessment: arrow heads pointing to the opening of a diverticulum located in the left inferior aspect of the buccal area.

**Figure 4:**
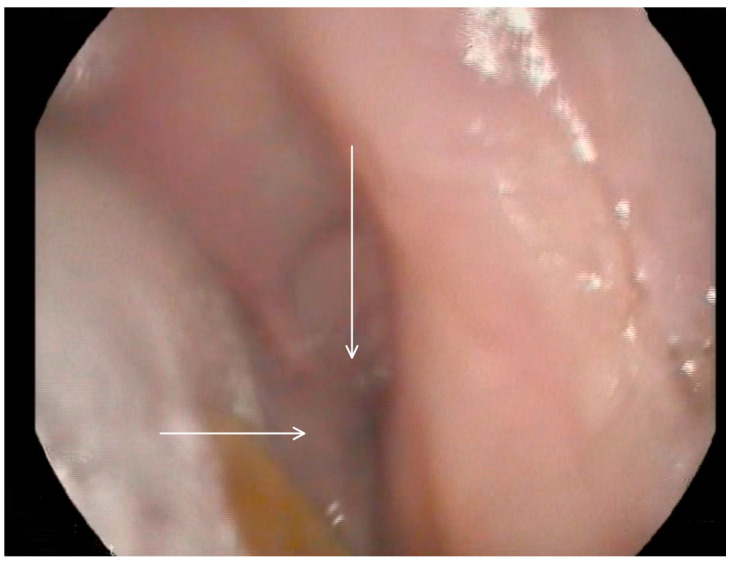
Selected still image captured during the FEES assessment: arrow heads pointing to the opening of a diverticulum located in the right inferior aspect of the buccal area.

**Figure 5:**
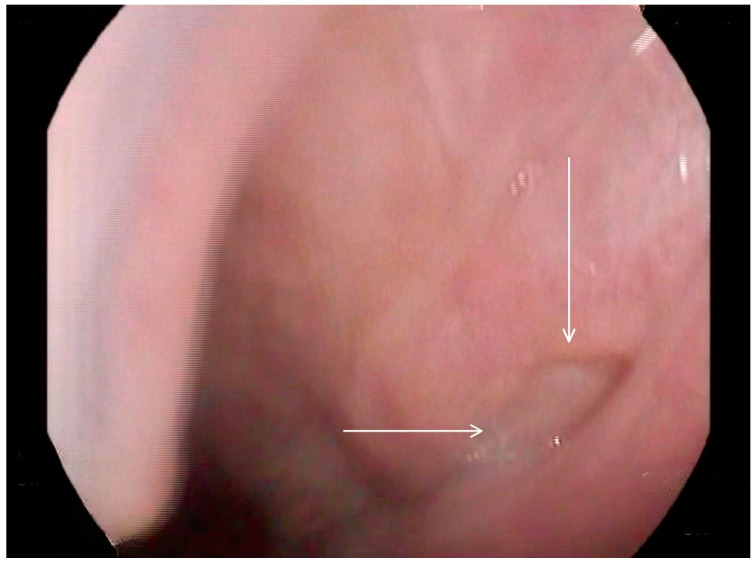
Selected still image captured during the FEES assessment: arrow heads pointing to the opening of a diverticulum located in the left superior aspect of the oropharynx area.

**Figure 6:**
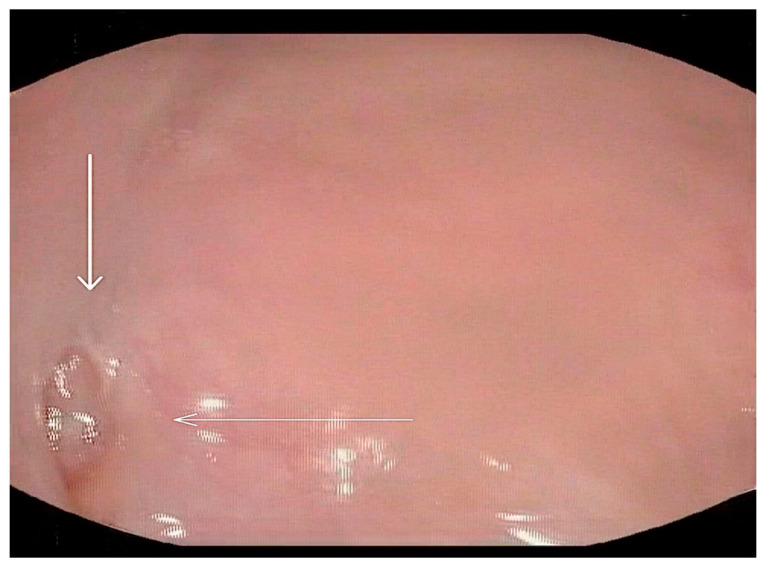
Selected still image captured during the FEES assessment: arrow heads pointing to the opening of a diverticulum located in the right superior aspect of the oropharyngeal area.

Following these findings, the ENT specialist reassessed the patient and confirmed the presence of bilateral diverticula in the buccal area and posterior pharyngeal walls, in alignment with the VFSS findings. The two diverticula were located on the right and left side inferiorly in the buccal area (Figure 3) and (Figure 4), and two were located on the right and left side superiorly in the oropharynx area. (Figure 5) and (Figure 6).

6. Management and Follow-up:

The patient was advised to modify the texture of thin liquids by using (Thicken up Clear) to alleviate the globus sensation and discomfort during swallowing. Despite the patient’s lack of commitment to attending follow-up appointments, he did attend one follow-up session after five months. At that time, he reported inconsistently following the swallowing recommendations by using the Thicken Up Clear. However, the patient reported a slight improvement in his swallowing and complained of feeling a globus sensation in his throat during swallowing. Given the persistence of symptoms, the SLP repeated the FEES study, and the results mirrored the initial findings, confirming the presence of oral and pharyngeal diverticula.

7. Diagnosis and Surgical Recommendation:

Based on these comprehensive evaluations, the patient was diagnosed with multiple diverticula in both the buccal and pharyngeal regions. Surgical intervention was recommended for definitive management, but the patient refused any invasive surgical or other therapeutic interventions.

## 3. Discussion

In this report, we describe a rare and interesting case of dysphagia associated with buccal and pharyngeal diverticulum diagnosed by a multidisciplinary team composed of speech and language pathologists, an ENT specialist, and a radiologist in Saudi Arabia. This study is considered rare as there have been few reports of diverticular disease in the oral and buccal cavities. However, it is possible that some cases were not reported due to misdiagnosis, the absence or presence of very few symptoms, or low morbidity of the lesion [2].

During the bedside evaluation, the results showed no complications during the swallowing process as the patient showed functional swallowing skills of both the oral and pharyngeal stages, nor were any signs of aspiration observed at that time. Additionally, an Oral Motor Examination (OME), which evaluates the structure and function of the face and oral cavity, including lips, tongue, jaw, soft palate, and hard palate, revealed no apparent motor or sensory facial deficits or oral cavity deficits, which did not align with the patient’s complaints. Consequently, an instrumental swallowing evaluation was conducted to investigate underlying structural abnormalities that the bedside swallowing evaluation may have missed. This evaluation, which included VFSS and FEES, provided comprehensive findings, identifying pouches in both the buccal and pharyngeal areas after swallowing. These findings are in agreement with the conclusion of similar studies suggesting that a barium swallow and/or an upper GI study are the best methods for visualizing and confirming pouches in the oral and pharyngeal areas [16,17].

In contrast, other studies relied on using instrumental radiography assessment to visualize and assess the presence of the pouches found in the oral and pharyngeal areas, thus providing an accurate differential diagnosis. CT assessments are typically appropriate for diagnosing lesions, determining the nature of the lesion (lytic or sclerotic), and examining bony structural changes and density. Meanwhile, MRI provides the characteristics of soft tissue extensions, which can evaluate and identify lesions at earlier stages [20,21]. In the case of the presence of oral and pharyngeal diverticulum, previous studies support using CT and MRI, which can identify a cavity filled with air or fluids, leading to the identification of the pouch [2]. However, in the current study, the CT and MRI studies showed unremarkable findings, with no cavities filled with fluids or air observed by the radiologist. This can be a limitation in the diagnostic capacity of CT and MRI in the cases of oropharyngeal diverticula. Conversely, only the barium and upper GI studies revealed the obvious imaging of the diverticulum found in the pharyngeal and oral cavities during the swallowing process [22,23], which aligns with similar studies that used these methods. Thus, the results suggested that barium studies and upper GI studies are the golden diagnostic tools for identifying pouches and/or diverticulum starting from the oral cavity down to the esophageal pathway.

The reported patient with the oral and pharyngeal diverticulum supports the observation that most cases tend to be asymptomatic, leading to a management plan where intervention may not be necessary. Unfortunately, in our patient’s case, he experienced severe difficulties in swallowing all the consistencies, which lasted for an extended period, along with a globus and looping sensation in his entire oral and pharyngeal cavity and sometimes in his face. These symptoms caused significant stress during mealtimes and eventually led to the loss of appetite and gradual weight loss. In addition, the given medical history of the patient is compatible with similar studies that suggested the risk factors that can contribute to oral and pharyngeal pouches, which are age, low fiber diet, and history of obesity, which all occurred in our case [1,6,7]. In terms of treatment, many studies have proposed surgical intervention using endoscopic tools under anesthesia to remove the lesion and obtain histopathological results, which in similar studies proved a positive outcome for cases with symptomatic oral and pharyngeal pouches as it completely resolved the patient’s symptoms [2]. However, in some cases, surgical intervention caused the disadvantages of enlarging the diverticulum, which has been documented during post-operation follow-ups [13]. However, the patient was not motivated to proceed with the recommended intervention plan and ultimately refused to proceed with any surgical treatment. This, in turn, halted the intervention cycle without any prospect of exploring its outcome.

## 4. Conclusions

In this research, it was essential to emphasize that various diagnostic assessment methods, including clinical bedside evaluations and radiography, can occasionally overlook the presence of pouches in the oral and pharyngeal cavities due to their rarity. It has been demonstrated that a VFSS and FEES continue to be the golden standard for the assessment of swallowing difficulties, specifically in symptomatic cases with an unremarkable BSSA. Based on the limited data in the literature, a pouch or diverticulum in the oral cavity has rarely been described. This current study provides clinicians with additional insight, which may lead to changes in their diagnostic protocols, ensuring every patient is provided with an accurate differential diagnosis.

Further research is essential to determine the physiology and etiology of oropharyngeal pouches, particularly in cases associated with dysphagia complaints in diverse contexts and across different populations. Such research is likely to result in a more generalizable finding using different diagnostic and management techniques.

## Data Availability

Due to the rareity of the case, data is unavailable due to ensure patient confidentiality and maintaincece of anonymity.
